# The Diagnosis Methods and Management Modalities of Maxillary Transverse Discrepancy

**DOI:** 10.7759/cureus.20482

**Published:** 2021-12-17

**Authors:** Nouf Bin Dakhil, Fahad Bin Salamah

**Affiliations:** 1 Orthodontics and Dentofacial Orthopaedics, King Saud Medical City, Riyadh, SAU

**Keywords:** segmental surgery, palatal expansion, skeletal maturity, transverse deficiency, maxillary discrepancy

## Abstract

Transverse deficiency of the maxilla (TDM) is the most common skeletal change that involves the maxilla. The craniofacial skeletal assessment as early as possible is critical, as the early diagnosis of TDM influences the effectiveness of treatment. Methods for treating TDM have been reported since the mid-19th century and continue to evolve. This article puts forward a literature review on the topic, investigating the diagnosis and management of TDM, as well as stability after surgical and nonsurgical interventions. We conducted a literature search using a logical combination of the terms “palatal extension,” “maxillary transverse deficiency,” “diagnosis,” and “management.” More recent approaches include three-dimensional imaging, which has allowed for accurate depictions of the craniofacial region to be examined, allowing for evaluation of the spatial relationships between the jaw elements. The success of nonsurgical management compared to surgical management depends on the growth stage of the patient. Unfortunately, data comparing the stability of surgical and nonsurgical management is still lacking. However, for surgical intervention, surgically assisted palatal expansion (SARPE) appears to be the appropriate choice, especially when a large expansion is needed.

## Introduction and background

There are three classifications of skeletal changes that involve the maxilla: vertical, horizontal, and transverse. The most prevalent among these are transverse changes [1], which often present solitary or in relation to other changes. Among such transverse changes, a reduction in transverse distance is the most common. This is also commonly referred to as a transverse deficiency of the maxilla (TDM) [2]. For surgical-orthodontic patients, this transverse dimension is frequently the most crucial plane of concern, despite it also being often the least recognized [3].

Vertical concerns are common, with patients often coming in with complaints like "teeth not touching in front" or “a big overbite.” Next, in the sagittal plane, a common complaint may be "chin not in the correct position" - something that could indicate either a skeletal Class II or Class III malocclusion. However, due to the limited visibility that patients have of their posterior maxilla, it is far less common that patients seek treatment for transverse malocclusion. Yet, as with many types of malocclusions, TDM significantly impacts the patients' jaw functions as well as appearance [3]. TDMs of this kind have been identified by orthodontists as a considerable proportion of all malocclusions [4].

To diagnose orthodontic abnormalities, which through prevention or treatment would measurably benefit patients, dental occlusion and facial growth must be assessed [5]. Treatment aims to reduce the potential for periodontal disease, enhance skeletal and dental stability, and improve the aesthetics of the smile [4]. Methods for overcoming transverse deficiencies of the maxilla have been reported as far back as the mid-19th century, with one such approach being lateral expansion at the mid-palatal suture of the maxilla’s bony halves [6]. Modern approaches to skeletal constriction of the maxilla include mid-palatal splitting via surgery in adults and slow or rapid expansion of the maxilla through orthopedic force in children and adolescents [7-9]. As adolescence trends to adulthood, the mid-palatal suture begins to fuse, and heavier orthodontic forces are required to impose skeletal expansion on the maxilla [9].

For diagnosis of TDM, it is therefore critical to perform the craniofacial skeletal assessment as early as possible, to ensure the effectiveness of treatment. Such need continues to motivate further development and evolution of maxillary transverse diagnostic tools [10]. This article puts forward a literature review on the topic, examining the diagnosis and management of TDM, as well as stability after surgical and nonsurgical interventions. A search of the literature was performed using a logical combination of the terms “palatal extension,” “maxillary transverse deficiency,” “diagnosis,” and “management.” The databases of PubMed® and Embase® were used to conduct the search. Thirteen studies were deemed relevant and discussed in this review.

## Review

Diagnosis

To effectively correct any dentofacial deformations that involve transverse deficiency, early and accurate diagnosis and treatment are imperative to stability [11]. Determining whether or what type of deficiency is present in the maxilla is the first step. TDM is more difficult to assess than vertical or sagittal discrepancy, as there are few changes in soft tissue that result from hypoplasia of the maxilla in the transverse direction [3,11]. Such soft tissue changes are much more prevalent when deformations are isolated to the anteroposterior or vertical [11]. Considerable literature exists regarding the approach and criteria for the diagnosis of maxillary deficiency. Accurate assessment has been successful using model analysis, clinical evaluations, radiographic measurement, and occlusograms [3].

For clinical evaluation, the form and symmetry of the maxillary arch, the palatal vault shape, occlusion, predominant breathing mode (i.e., oral or nasal), and buccal corridor width when smiling were all included in assessment. Manifestations indicating the presence of transverse deficiency in the maxilla include hollowing of the paranasal region, excessive width of the buccal corridors, nasolabial fold deepening, or narrowing of the alar bases. Soft tissue changes associated with TDM are minimal, which may complicate the diagnosis. Thus, severe crowding, rotation, or buccal/palatal displacement of the teeth, crossbite (uni- or bilateral), high palatal vault, and hourglass- or V-shaped occlusions are considered among the main manifestations. Mouth breathing is one of the etiologies of TDM. Thus, the aforementioned findings are occasionally observed in otolaryngology practice. Patients with TDM should be evaluated for the possibility of mouth breathing, and must be referred to the appropriate specialty as indicated. Dentofacial deformities associated with TDM may include vertical maxillary excess, relative mandibular prognathism, apertognathia, and repaired cleft palate. These are visual indicators that allow a clinician to make a first determination regarding transverse deficiency [11-13]. Mandibular shift upon closure should be assessed. Lateral chin deviation may be noted in a frontal facial examination, and if so, its root cause needs to be identified. This could be due to a functional shift from centric relation, or actual skeletal asymmetry. Uncertainty regarding lateral shift should be allayed via temporary disarticulation of the occlusion for one to two weeks, followed by a re-examination. This can be done with a bite plate. Compliance of the patient may be at issue here, particularly so for younger patients. In this case, a fixed (Hyrax) expander can be inserted and mildly activated, disarticulating the occlusion and allowing a lateral shift to be inspected. It is important to inform the involved parties, whether patient or parent, that definitive treatment may not be possible until the lateral shift has been identified or ruled out. If a lateral shift is found to be absent, then unilateral crossbite and chin asymmetry serve to indicate true unilateral skeletal asymmetry [14].

The form and shape of the arch should be evaluated through the use of study models. This will allow for specific measurements to be made that evaluate the transverse deficiency of the maxilla. A number of authors have put forward indexes for such lateral measurements. Pont, Linder-Harth, and Korkhaus provide the most common among these [15]. While diagnosis of TDM is aided by such indexes, they cannot be relied on entirely, as they are population-specific [16]. Arch symmetry and transverse tooth inclination variability must be further analyzed via study cast. It is possible to see bilateral crossbite occlusion without any chin asymmetry or lateral shift. If this is found, the patient’s study casts and knowledge of the sagittal relationship allow for classification of such a transverse discrepancy. Sagittal and transverse interarch relationships change with respect to one another. Examination of patient study casts is again required in order to determine whether the transverse deficiency is absolute or relative. If the proper transverse cusp-fossa relationships in centric relation are not exhibited by the posterior teeth, then the transverse discrepancy is said to be relative when the posterior teeth will properly occlude (if tooth alignment were correct) when the canines are placed in Class I occlusion in this case [14,17]. Some Class III malocclusions, for instance, will involve a posterior crossbite that is eliminated when casts (arches) are articulated into a Class I canine relationship. Again, this transverse discrepancy would be classified as relative. However, if after a Class I canine relationship is articulated in the casts, a crossbite is still present, the transverse discrepancy is classified as absolute [14]. In the case of an absolute transverse discrepancy being present, the origin (skeletal or dental) and magnitude of the discrepancy are determined via study casts. Posterior dental compensations in the cast should be investigated first. These will present as variations in the permanent first molars’ transverse axial inclination - most frequently, excessive torque on the maxillary buccal crown or the mandibular lingual crown relative to the frontal plane. [14].

A gross estimation or a measurement using the American Board of Orthodontics (ABO) measuring gauge will suffice. Placing an ABO gauge across left and right first molars defines a transverse occlusal plane. The gauge should contact both the buccal and lingual cusps if the molar being investigated has a transverse axial inclination perpendicular to said plane. Displacement from this perpendicular inclination will demonstrate that the lingual or buccal cusps are displaced from the transverse occlusal plane. The ABO gauge allows for 1mm incremental estimations. Average width molars have 5-6mm of distance between the buccal and lingual cusps. A 1mm displacement from the transverse occlusal plane is therefore approximately 10^o^ of buccolingual inclination. The buccolingual tilt of mandibular molars can also be evaluated using this method [14]. Authors of Iowa Facial Growth Study investigated molar inclination in those with normal sagittal and transverse occlusion [18]. Subjects aged 7-26 years old were included and their molar inclination measured. An average of 10^o^ +/- 4^o^ of buccal crown inclination was found in the maxillary molars of subjects aged seven [18]. The same subjects had 10^o^ +/- 5^o^ of lingual crown inclination in the mandibular molars [18]. Later growth was found to result in mandibular and maxillary molar inclination change more perpendicular to the transverse occlusal plane [14]. To differentiate between dental and skeletal discrepancies, a method of counting teeth has been suggested. [19]. If two or more posterior teeth are involved in the crossbite, the discrepancy is said to be skeletal [20]. While useful for its simplicity, the rule is also misleading. Even with no posterior teeth in crossbite, severe skeletal transverse discrepancies can be present, masked by posterior dental compensations. If improvement of the posterior transverse interarch relationship results from upright of the molars in the cast (i e , removal of the transverse compensations), then a dental origin is likely for the transverse discrepancy. Dental movement alone could then be applied as treatment. If in the same case there is a worsening of the posterior transverse interarch relationship, then a skeletal origin for the discrepancy is much more likely [14].

For evaluating midpalatal suture ossification, Lehman et al. [21] recommend as an essential tool an occlusal or palatal radiograph. The superposition onto the midpalatal suture or other bony structures and an inability to effectively visualize the posterior intermaxillary suture, however, make this an unreliable method overall. Obliteration of the intermaxillary suture has been shown by histological study to be more common in the posterior region. Studies have also demonstrated that the midpalatal suture offers little resistance to expansion, further putting the value of an occlusal radiograph to evaluate it into question [22-24]. For identifying and evaluating transverse discrepancies of a skeletal origin in the mandible and maxilla, Betts et al. [1] suggest posteroanterior (PA) cephalograms as the surest and most accessible approach. Ricketts [25] describes a set of cephalometric landmarks that allow Betts et al. [25] to define two quantifications of transverse deficiency in the maxilla. These are: maxillomandibular width differential and maxillomandibular transverse differential index. Criticisms of these methods do exist, however. For instance, the bony landmarks used to measure maxillary and mandibular transverse discrepancy have a great degree of separation from the apical and dentition bases [26]. The Rocky Mountain analysis, developed by Ricketts [25], was intended to establish relative norms between specific radiographic landmarks with further measurements required for analysis of maxillary/mandibular transverse discrepancy. The frontolateral facial lines and the effective mandibular and maxillary widths can be determined using said landmarks. These widths can be defined as follows: maxillary width is the width between the points jugale left (JL) and jugale right (JR), while mandibular width is the width between the right antegonial tubercle (AG) and the left antegonial tubercle (GA). The frontolateral facial lines are the lateral lines constructed from orbitale right (OR) and orbitale left (OL) to the points AG and GA, respectively. To quantify the transverse maxillary discrepancy, these cephalometric landmarks can be used to first calculate the maxillomandibular transverse differential index and the maxillomandibular width differential itself. The latter of these is simply the displacement (in millimeters) measured from the frontolateral facial line to JL and JR, respectively, along a line from the frontolateral facial lines through JR and JL. This is an independent measurement made on each side and compared to the normal value of 10 +/- 1 5 mm. A value greater than 10mm indicates the existence of a transverse discrepancy between the mandible and the maxilla. The total transverse deficiency is quantified by summing the measured amounts on both sides in excess of 10mm. This is a useful method for illustrating the total discrepancy and identifying which side contributes a greater deficiency. When mandibular asymmetry is present, though, this technique may be misinterpreted, as it does not effectively determine in which jaw the discrepancy is present [11].

The maxillomandibular transverse differential index, on the other hand, takes the actual measured maxillomandibular distance and subtracts it from the expected difference for the patient’s age. The expected difference is the age-specific expected AG-GA distance minus the age-appropriate expected JR-JL distance, while the actual maxillomandibular difference is the actual AG-GA measurement minus the actual JR-JL measurement. A maxillomandibular transverse differential index greater than 5mm in an adult patient indicates that surgical expansion is necessary. This method not only quantifies the total discrepancy, it also allows for a determination of which jaw is excessive or deficient. This is because actual values can be compared with normal values, though it is important to note that “normal values” have only been suggested for Caucasians; therefore, they should not be considered for patients of other races [11].

Traditional two-dimensional (2D) skeletal structure imaging is known to suffer from technical limitations that affect landmark placement accuracy. This, in addition to inexperience among practitioners in identifying them, results in considerable errors in landmark identification [27-29]. Only asymmetries can be diagnosed by PA radiographs, no other transverse problems [14]. More recent approaches include 3-dimensional (3D) imaging, which have allowed for accurate depictions of the craniofacial region to be examined, allowing in turn for direct visual evaluation of the spatial relationships between elements of the jaws [30]. Cross-sectional evaluation of 3D representations of the apical bases are possible with cone-beam computed tomography (CBCT) scans. A clinician can use these images to make a more accurate and more detailed assessment of asymmetries and the nature and location of discrepancies [26] While specific, localized transversal radiographic cuts of CBCT scans is of clinical importance for assessing areas of interest, it also has considerable diagnostic potential in regard to the craniofacial transverse dimension. CBCT imaging availability is increasing, which makes it a timely and beneficial question to determine its ability to improve TDM diagnostics or whether it is only useful for improving the precision of landmark location. Before advocating for greater use of CBCT and the ionizing radiation associated with it, validation of its clinically significant reliability and accuracy should be obtained [10]. Technique-related factors, such as standing position, should be taken into the account when the measurements include temporomandibular joints spaces.

Management

In children and early adolescents, orthodontic expansion as applied to the maxillary dental arch dates back over a century in the literature, with Angell [31] and later Clavero-Juste [32] and Korkhaus [33] reintroduced the technique in the US in the 1950s, bringing a modern approach to these expansion techniques, which went on to gain popularity, thanks to Haas and others [3,34,35]. There are many techniques for expansion of this type, including rapid expansion, slow expansion, alternating constriction and expansion, archwire expansion, and temporary anchorage device-supported expansion, most recently [3]. Rapid expansion has been used for TDM associated with mouth breathing. However, it is very important that if an ongoing etiology of TDM is identified, such as mouth breathing or thumb sucking, it has to be addressed as much as possible before initiating the orthodontic treatment.

**Figure 1:**
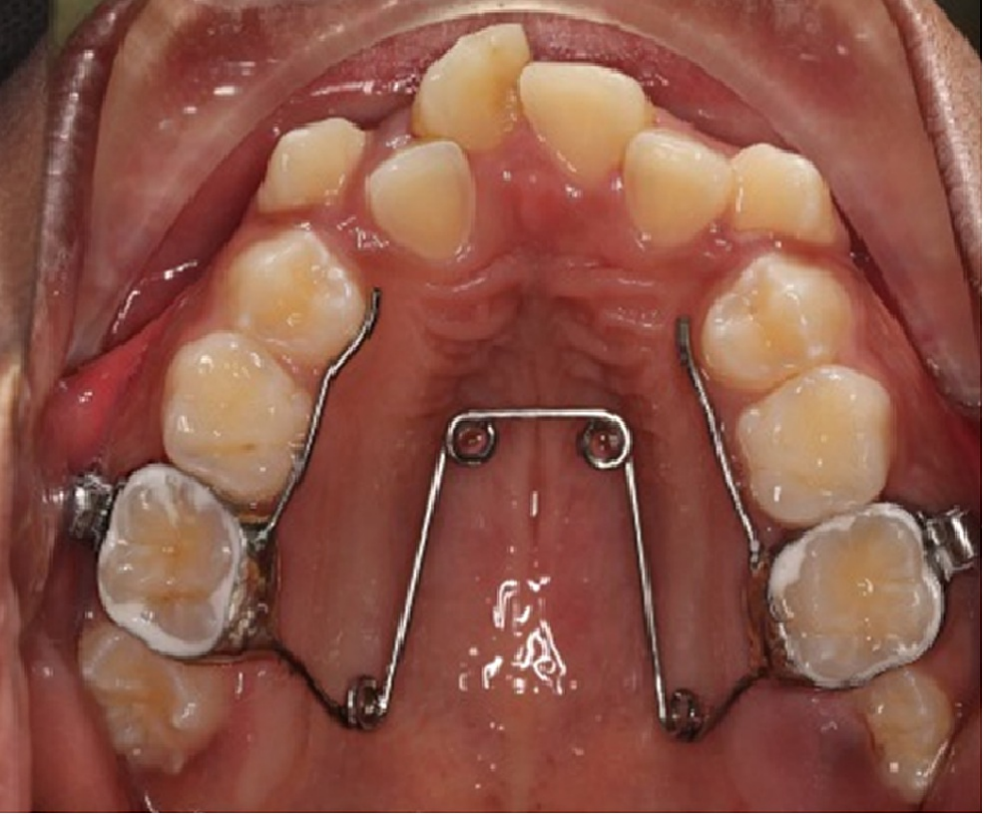
Quadhelix expander

There are several different techniques that can be used to successfully widen the dental arch. It should be noted, however, that any approach will be limited in how much it can widen without widening the basal bone as well. Orthodontic brackets with a wide archwire are among the simplest dental expansion devices. Quadhelix (Figure 1), cross-arch elastics, and transpalatal arches (TPA) are some other tools [3]. TDM correction is among the reasons why it is recommended that every child receive an orthodontic examination by seven years of age. Several skeletal malocclusion can also be detected early, e.g., in Class II patients with >7 mm of overjet or in Class III patients with <-1 mm of overjet. It is possible for both such patients to have TDM. There are a variety of benefits to recognizing and correcting this deficiency early [3]. The prevention of asymmetric growth in the presence of bilateral crossbite with functional shift and the reduction in or elimination of the necessity for surgical correction are two primary benefits. A greater arch perimeter to accommodate future teeth alignment, sagittal malocclusion improvement, and potential airway improvements are among some of the possible secondary benefits [34]. It is most easy to accomplish orthopedic expansion before closure of the midfacial sutures and cranial base has taken place. The first plane in which growth ceases has been shown to be the transverse dimension, at the inter-spheroidal and inter-ethmoidal sutures in particular. Both are found by McNamara and colleagues [36]. to close before nine year of age. At this point, an increasing amount of dental and decreasing amount of skeletal expansion continue, until late adolescence, at which point there is little to no skeletal expansion [37]. With increasing age, the circum-maxillary sutures progress along a similar path of increasing complexity and decreasing patency [38].

**Figure 2:**
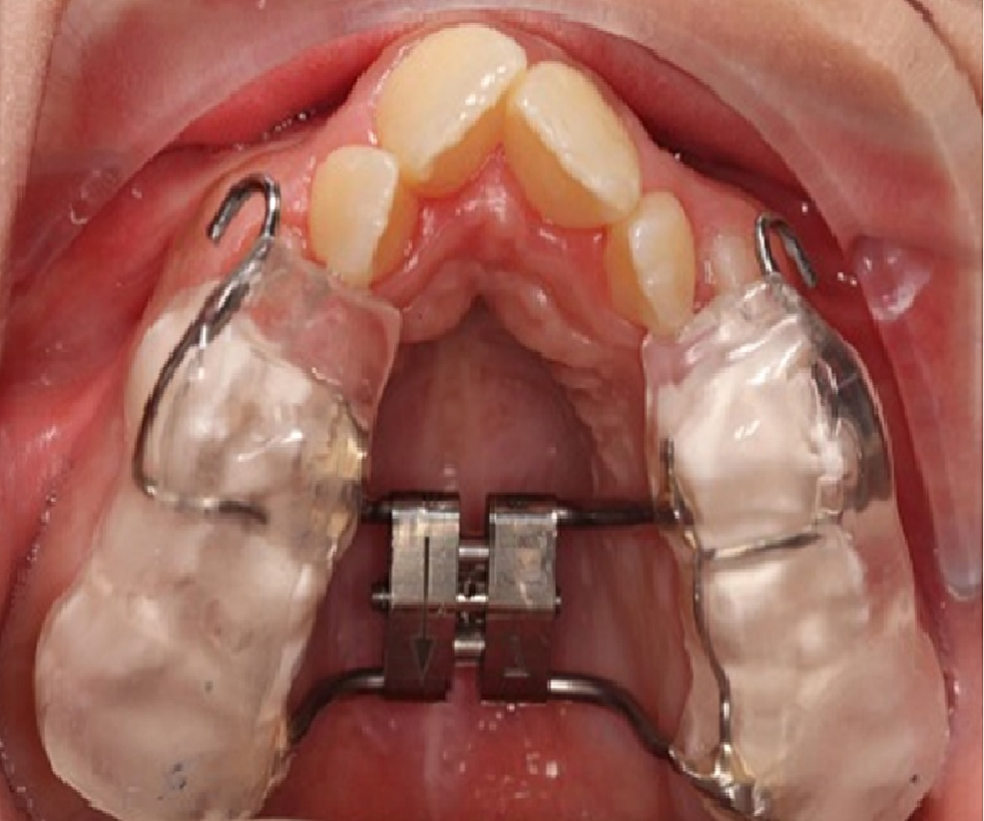
Bonded palatal expander

**Figure 3:**
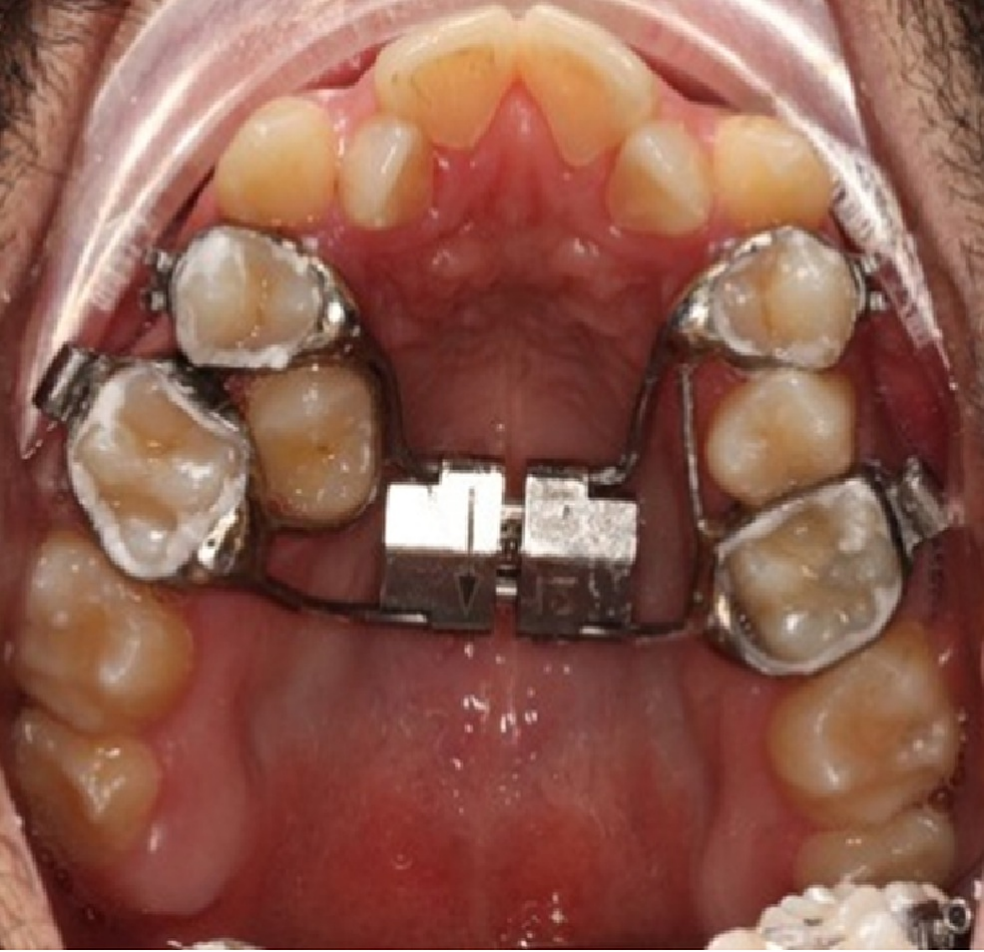
Hyrax expander

Assessments of skeletal maturity have been proposed, among them cervical vertebral maturation, hand-wrist radiographs, and more recently using CBCT to assess maxillary sutural maturity [38]. The appliances used to carry out orthopedic expansion are generally categorized into two types: tooth- plus tissue-borne expanders and tooth-borne rapid palatal expanders, both of which can be bonded (Figure 2) or banded to teeth. There remains a debate as to which of these two yields greater skeletal expansion, but both have been shown to be successful [17,39]. Slow activation protocols mandate one turn every two to three days, while rapid protocols see closer to one to two turns each day. Proffit et al. [9,40] claim that similar skeletal or dental expansion results at the end of the expansion period. The following are indications of orthopedic skeletal expansion: the presence of crossbite, narrow maxillae in Class II patients, Class III patients for whom simultaneous expansion and protraction is considered, and non-skeletally mature (i.e., growing) patients [3]. The first descriptions of rapid maxillary expansion (RME) were made in 1860-61 by Emerson Angell and further developed and promoted by Haas. The primary purpose of RME is to widen the maxillary arch, but its effects are not limited to the maxilla, as it involves 10 other craniofacial bones [41]. RME advocates claim that it results in maximum skeletal movement with minimal dental movement (tipping) [42]. Heavy, rapid force on the posterior teeth does not allow for tooth movement to occur in time, transferring the force instead to the sutures. Once the appliance is delivering a force in excess of the limit for orthodontic tooth movement, the sutures are opened and the teeth undergo minimal movement relative to their skeletal support. The periodontal ligaments are compressed, the alveolar process is bent, the anchor teeth are tipped, and the midpalatal suture and eventually all other maxillary sutures are opened [43]. RME appliances may banded or bonded. For the former, bands are used to attach the appliance between the maxillary first molar and the first premolars. There is no palatal coverage, making the banded appliances a more hygienic approach. Banded RMEs can be of two categories: tooth and tissue borne (e.g. Hass expander) or tooth borne (e.g., Hyrax expander) (Figure 3) [43]. There is less tissue resistance produced on the circum-maxillary structures by slow maxillary expansion, improving intermaxillary suture bone formation and theoretically eliminating or mitigating RME limitations. If an adequate retention period is given, greater post-expansion stability has also been found to result from slow expansion [44-47] This approach allows a constant physiological force to be applied until the necessary expansion has been achieved. A typical appliance for slow maxillary expansion is a Quadhelix [43].

In skeletally mature patients (i.e., non-growing), maxillary dental arch expansion can only be achieved through dental movement. Adult patients who exhibit skeletal transverse deficiency may need tooth movement that extends further than the alveolar bony base of the maxilla. This can result in anchor teeth tipping, extrusion, root resorption, alveolar bone bending, periodontal membrane compression, relapse, fenestration of the buccal cortex, palatal tissue necrosis, pain, inability to open the midpalatal suture, and expansion instability [22, 48-59].

Surgically assisted orthodontic palatal expansion (SARPE) or segmental surgery can be used instead to alter the skeletal transverse dimension of adult patients. The midpalatal suture was typically considered the primary area of resistance with respect to orthodontic expansion. Initial reports on surgical intervention to assist palatal expansion therefore included midpalatal splitting [3]. Lines [24], in 1975, and Bell and Epker [23], in 1976, found conclusively that the zygomaticomaxillary, zygomaticotemporal, and zygomaticofrontal suture areas were the primary sites of resistance to maxillary expansion, not the midpalatal suture. Wertz [22] went further to propose that in fact the zygomatic arches were the source of the resistance. A variety of osteotomies have been designed to account for these varied sources of resistance, meant to weaken those particular areas to allow for greater expansion [26]. It is rare for transverse skeletal deficiency to occur if no vertical and/or horizontal discrepancy co-exists alongside it. Distraction should be taken into account whenever correction of maxillary constriction is the sole objective. The maxillary arch may be widened, though, as part of a treatment plan involving many other corrective orthognathic surgical procedures [3]. Either segmental osteotomies or SARPE may be used to achieve surgical correction of TDMs. The former is preferred in situations where all maxilla-mandibular discrepancies are to be treated with a single surgical procedure. This is because segmental osteotomies allow for vertical and sagittal reposition of the maxilla to be performed at the same time as a correction of TDM. Alternatively, with SARPE, TDM correction is carried out as a first step, and any further maxilla-mandibular repositioning in other planes must be done as a separate surgery. SARPE has been recommended by Bailey et al. [60] for isolated transverse deficiencies in patients for whom there is no indication of orthopedic maxillary expansion (OME), or for patients with narrowing of the maxilla (asymmetric or unilateral). While the use of SARPE may seem limited by this account, the long-term stability, morbidity, and psychological impact of a two-stage vs. one-stage procedure needs to be taken into account. Advocates of SARPE have pointed out that it may also be possible for orthopedic forces to be applied post-SARPE, when the two maxillary halves are loosened. Sagittal and vertical discrepancies could therefore be corrected without the need for a second surgery. Prognosis of such attempts is uncertain, and so they have not been routinely used [26]. It is a matter of some debate still among surgeons and orthodontists what the indicators are for SARPE [3]. TDM is difficult to accurately diagnose, despite maxillary expansion itself being a common requirement. In cases of skeletally mature patients with constricted maxillary arches, indications for SARPE have included: 1. Correction of posterior crossbite, increasing maxillary arch perimeter, or when there are no further surgical jaw movements in the treatment plan; 2. Widening of the maxillary arch as a preliminary procedure for other orthognathic surgery, to mitigate risk, inaccuracy, and instability; 3. Providing more space for maxillary dentition crowding when extraction not indicated; 4. Widening of maxillary hypoplasia in palate cleft patients; 5. Reducing wide black buccal corridors in the smile; and 6. Overcoming suture resistance in the case of OME failure [3,61,62].

In many patients, it is obvious that a large degree of expansion is required (>7 mm). A tooth-borne expander must be in place before the patient goes into the OR, if such an appliance will be used. In other cases, though, the scale of transverse deficiency may not be as readily clear. In the mandibular arch, the Curve of Wilson, and in the maxillary arch, the Curve of Monson, can be examined. A practical approach if uncertainty is still present involves placing a full fixed mandibular orthodontic appliance to upright and level the Curve of Wilson. New scans or models can then be made, and the transverse deficiency can be reexamined to ensure that the treatment plan encompasses the full extent of what is necessary. Significant Class II or Class III deformities may be among the confounding variables. To visualize the true transverse discrepancy, models should always be investigated in the final expected sagittal occlusion. If SARPE is indicated, the case can be aided significantly with the following procedural techniques. First, the teeth roots that are adjacent the osteotomy should be clearly divergent, or else they should be actively diverged by the orthodontist in order to aid in the surgical cut. Subtle archwire bends or adjustment of bracket orientation during initial bonding can both be done quite simply to achieve this. In either case, no maxillary expansion should be performed. The orthodontist should only work to develop root separation/divergence. Finally, it should be considered that the space between crowns is not what is most important; inter-radicular space is. Open coil springs should therefore be avoided, as they typically result in a greater degree of root convergence. After the required divergence has been achieved, the archwire should be removed completely. This should be done the same day as the surgery, or else it should be sectioned before or during SARPE, ensuring that both maxillary halves. and the relevant dentition are not resistant to expansion [3].

Spacing or limited crowding are ideal in a patient, as they allow for alignment of the teeth before expansion. Interproximal reduction (IPR), can usually be used to address limited crowding. This can even be done before alignment if natural contact points are accessible, leading to the lowest probability of dental expansion. The teeth can also be aligned initially, if the natural contact points are not accessible. IPR is then performed to reverse any accidental or unintended expansion. The optimal alignment is achieved if, after segmentation, the teeth will fit the opposing arch. When dental arches are crowded, there will be no space available for teeth alignment. Extraction may therefore be necessary in cases of significant crowding. Although this adds presurgical orthodontic time to the procedure, it is nonetheless the ideal approach for crowded dental arches, as not only does it result in space to align the teeth, it also produces the sagittal occlusion that is desired. Space can also be used as inter-radicular space for segmental osteotomies [3]. The patient will return to the orthodontist after approximately six weeks to allow for hard callus formation and primary stability. A heavy continuous stainless steel archwire should be used (17X25 in and 18 slot; 19X25 or 21X25 for 22 slot) [3].

Stability after treatment

There is no in-depth investigation in the literature of the long-term stability or relapse risk of SARPE. Surgical expansion is found in most cases to have a higher degree of stability than does OME [21,63-65]. Retention is said by some authors to be unnecessary for SARPE, so that the orthodontist is able to start treatment with no holding phase necessary [65]. Other recommendations in the literature vary in recommending a post-expansion retention period of 2-12 months [62,63,66-69]. SARPE relapse rates are reported to be around 5-25% [65,70-72]. The relapse rate of OME can be significantly higher, with some reports placing it as high as 63% [7,73,74].

The high relapse rate of OME is associated with its patient demographics, most of whom are skeletally advanced and for whom the procedure is not predictable nor is it stable. Berger et al. [71]. compared SARPE and OME in an age-appropriate sample. Subjects were aged six years to 12 years for OME, while SARPE subjects ranged from 13 years of age to 35 years. There was no difference reported in stability between the two methods in this study. Relapse was not quantified for either group [26].

Most of the literature on SARPE does include a recommendation that clinicians be aware of the possibility for relapse, but its incidence is reported to be low. There is little to suggest that overexpansion is necessary with SARPE, but some do cite it, particularly for bone-borne appliances [2,21,63]. Relapse was still reported to be extremely low in such cases [70,75].

Proffit et al. [76] carried out a review of postoperative stability in orthognathic surgical patients, finding that the least stability offered by any means of maxillary expansion is when it is done through segmental surgery. However, this review was carried out in 1996, and the surgical techniques and postoperative control have advanced considerably since then, undoubtedly leading to improved transverse stability. Indeed, the same authors published an improvement in an extension of their earlier work in 2007, following the development of rigid fixation [40]. Woods et al. [40] found that segmental surgery was found to be unstable especially when large amount of expansion is needed (> 8mm), while for SARPE the relapse was found to be low.

Limitations of this review include those related to non-systematic reviews. It is possible that some relevant studies might not have been examined. Nonetheless, it appears that literature about TDM is still limited, and more studies addressing this topic are needed.

## Conclusions

TDM is more challenging to assess than vertical or sagittal discrepancies. Appropriate diagnosis and treatment planning is crucial in the treatment of TDM. Such need continues to motivate further development and evolution of diagnostic tools. Management modalities are grouped into two main categories: orthodontic treatment alone or combined surgical and orthodontic treatment, depending on the growth stage of the patient. For surgical intervention, segmental surgery was found to be unstable especially when large expansion is needed (> 8mm), while for SARPE the relapse was found to be more stable. Therefore, SARPE appears to be the appropriate choice when surgical intervention is planned. Nevertheless, more investigations regarding the stability of each approach are still needed.
